# Iron-Enriched Nutritional Supplements for the 2030 Pharmacy Shelves

**DOI:** 10.3390/nu13020378

**Published:** 2021-01-26

**Authors:** Giulio Verna, Annamaria Sila, Marina Liso, Mauro Mastronardi, Marcello Chieppa, Hellas Cena, Pietro Campiglia

**Affiliations:** 1 Department of Pharmacy, University of Salerno, 84084 Fisciano, Italy; 2 National Institute of Gastroenterology “S. de Bellis”, Institute of Research, 70013 Castellana Grotte, Italy; a.sila@outlook.it (A.S.); marinaliso@libero.it (M.L.); mauro.mastronardi@irccsdebellis.it (M.M.); marcello.chieppa@irccsdebellis.it (M.C.); 3 Laboratory of Dietetics and Clinical Nutrition, Department of Public Health, Experimental and Forensic Medicine, University of Pavia, 27100 Pavia, Italy; hellas.cena@unipv.it; 4 Clinical Nutrition and Dietetics Service, Unit of Internal Medicine and Endocrinology, ICS Maugeri I.R.C.C.S, 27100 Pavia, Italy

**Keywords:** iron, nutrition, IBD, iron deficiency, algae, superfoods

## Abstract

Iron deficiency (ID) affects people of all ages in many countries. Due to intestinal blood loss and reduced iron absorption, ID is a threat to IBD patients, women, and children the most. Current therapies can efficiently recover normal serum transferrin saturation and hemoglobin concentration but may cause several side effects, including intestinal inflammation. ID patients may benefit from innovative nutritional supplements that may satisfy iron needs without side effects. There is a growing interest in new iron-rich superfoods, like algae and mushrooms, which combine antioxidant and anti-inflammatory properties with iron richness.

## 1. Introduction

Iron is an essential metal for human physiology, and it is involved in several cellular metabolic reactions including oxygen transport [1]. Most living organisms have developed different strategies to acquire, store, and recycle iron [2,3]. In humans, inorganic iron is reduced in the gut lumen and absorbed by divalent metal cation transporters expressed by epithelial cells in the duodenum [4]. A different source of iron is represented by heme-iron, which is directly absorbed, mainly in the large intestine, through specific receptors [5]. Once absorbed, it is transported into the bloodstream, bound to transferrin, and stored in the liver [6]. Among these two different pathways, the main dietary iron uptake is heme-iron, derived from meat and fish; while non-heme iron is derived from plants, vegetables, fruits [7,8], and iron-fortified foods [9,10]. Some nutrients can also influence iron absorption: vitamins like ascorbic acid enhance its absorption, as well as prebiotics, probiotics [11] and symbiotics [12], while polyphenols like phytate reduce its bioavailability and absorption; [13] also, calcium can inhibit iron absorption, even when it is administered as Ca salts or in dairy products [14].

Iron levels are tightly balanced because both iron-deficiency and iron-overload affect human health, impairing oxygen transport [15,16], inducing tissue damage particularly in the liver [1], and impairing inflammatory responses. The effects of iron availability on the immune cell inflammatory capabilities are less likely considered. The physiological iron concentration acts as a powerful support for inflammation [17] while its reduced or increased concentration can hinder an effective immune response [18,19]. Every day small quantities of iron are lost through enterocyte shedding and sweat, but under normal conditions, they are compensated by habitual dietary intake [20,21]. Because iron is necessary for red blood cell formation, a reduced concentration in circulating and stored iron may lead to the development of anemia [22].

Iron deficiency anemia (IDA) is a threat in many developing countries [23]; helminths and other parasite infections may cause anemia [24,25,26,27]. IDA is often associated with unbalanced nutritional regimes, rich in natural phytochelators, with poor heme content [28], and/or excessive blood loss. Not surprisingly, IDA is frequent in women (as a consequence of menstrual bleeding) [29,30] and children [31].

Iron deficiency (ID) has many causes, including reduced intake, impaired absorption, blood loss, caused by menstruation, IBD (Inflammatory Bowel Diseases) or injuries [32,33].

Inflammation can influence iron absorption as well. Chronic intestinal inflammation and, notably, IBDs including ulcerative colitis (UC) and Crohn’s disease (CD) damage the intestinal mucosa and are characterized by frequent blood losses [34]. During flare episodes, which occur more frequently in UC than CD patients, small ulcerations of the mucosa can cause IDA in 36–76% of patients [35,36,37,38,39]

In IBD patients, the clinical manifestations of IDA are secondary to insufficient dietary intake, mucosal ulcerations with blood loss, and anemia of inflammation, caused by inadequate transepithelial iron absorption in the gut. Furthermore, the reduction of iron absorption may also cause IDA in IBD patients.

**Figure 1:**
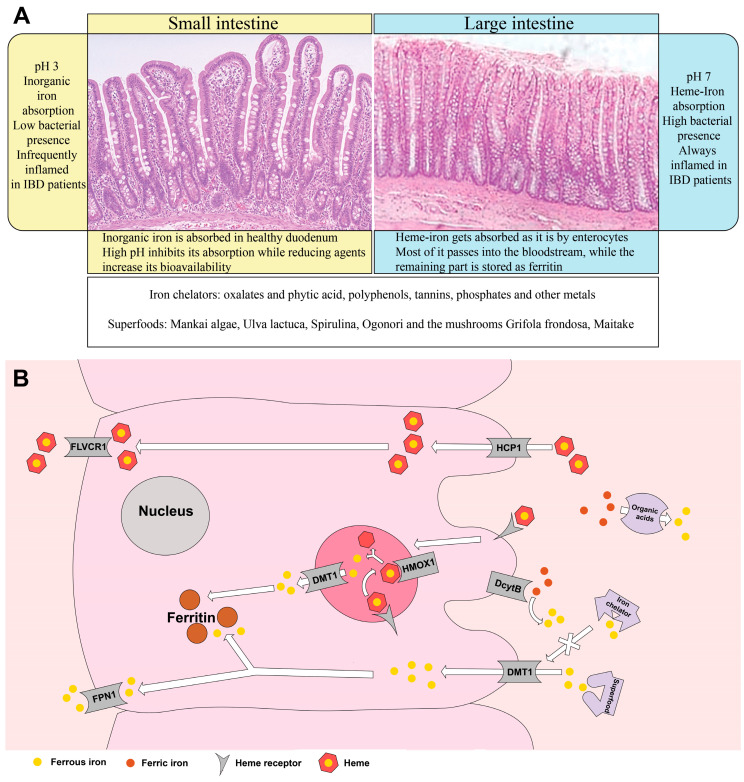
(**A**) Influence on iron absorption of pH, intestinal tract, and nutrients. Many nutrients and inorganic metals influence iron bioavailability and can increase or decrease its absorption; superfoods are often rich in iron, proteins, and vitamins. A Mediterranean diet supplemented with Mankai smoothies, for example, provides the right amount of daily iron. Superfoods also show antioxidant, immunomodulatory, and anti-inflammatory properties. (**B**) Iron absorption is influenced by food contents. Heme-iron is easier to absorb and can be directly absorbed or dissociated into ferrous ions and porphyrinic ring. Ferric ions need to be reduced to ferrous ions either by organic acids in the intestinal lumen or by DcytB and then they can be transported by DMT1 in the enterocyte and into the bloodstream by ferroportin. Intracellular iron can be also stored as ferritin. Iron chelators “steal” iron ions from the cells as opposed to superfoods that provide and “liberate” iron ions to the cells. (FLVCR1: Feline Leukemia Virus Subgroup C Receptor-Related Protein 1; HCP1: Heme Carrier Protein 1; DMT1: Divalent Metal Transporter 1; HMOX1: Heme Oxygenase 1; DcytB: Duodenal Cytochrome B; FPN1: Ferroportin 1).

Inorganic iron is absorbed mostly in the duodenum and in the proximal ileum mainly due to the low pH, which keeps iron soluble; different pH values along the gastrointestinal tract influence iron absorption (Figure 1A). In the duodenum and the first tract of the jejunum, pH is moderately acid, while in the distal tract of the small intestine as well as in the colon, it becomes progressively more alkaline. Of notice, inflammation can change the luminal pH and, consequently, change host iron absorption capacity.

Furthermore, inflammation induces hepcidin activity due to inflammatory cytokines release (e.g., IL-6). Hepcidin binds to ferroportin on enterocytes, thus causing its internalization and lysosomal degradation, leading to intracellular iron sequestration [40].

Moreover, there are also other chronic diseases like obesity and lifestyle factors as high levels of physical activity that may respectively impair iron absorption and increase Fe requirements, due to the increased erythropoietic drive caused by regular exercise [41]. Obesity as well as noncommunicable diseases (NCDs) [42] and diabetes, often show signs of silent inflammation that, in turn, influence iron absorption. Obesity has showed increasing morbidity in recent years and showed signs of latent inflammation that increase serum hepcidin and, consequently, reduce serum ferritin saturation [43,44]. It is worth noting that anemia is correlated with obesity in young patients; low-grade inflammation was observed to be a risk factor for ID even in schoolchildren [45]. Type 1 and 2 diabetes are widespread pathologies that appear to be associated with ID; the coexistence of high glucose levels and inflammatory cytokines impairs or reduces intestinal iron absorption through enterocyte iron transporters downregulation [46,47].

Foods rich in antioxidants (namely fruit and vegetables) can help reducing the inflammatory burden on intestine [48] as well as on adipose tissue [49], but, unluckily, they might reduce iron absorption too. Although citric acid and Vitamin C, mainly found in fruits, enhance iron absorption, plant food contains various ‘anti-nutrient’ compounds including lectins, oxalates, phytates, phytoestrogens, and tannins, that are thought to restrict bioavailability of key nutrients, as iron [50]. Nonetheless many studies concluded that these foods, also known as superfoods [51], have health promoting effects and provide significant reduction in chronic disease risk, attributable to the synergistic actions of these anti-inflammatory compounds [50].

Therefore, the correct provision of those superfoods, which combine anti-inflammatory, antioxidant, and high iron content is crucial in the treatment of chronic diseases and fight ID [52].

In view of the importance of iron for human health, considering its metabolism and all factors affecting its absorption or reducing its intake, researchers are trying to find new nutritional strategies including iron rich superfoods as dietary supplements that may soon become recommended for ID treatment. The focus of this review is to discuss about the nutritional sources of iron, foods that can reduce its absorption and superfoods rich in iron that can be used as nutritional supplements.

## 2. Nutritional Sources of Iron

Every day, approximately 25 mg of iron are needed by our body for its correct homeostasis, most of them are required for hematopoiesis. A large part of the iron requirement is sustained by the recycling process of aged red blood cells, with only less than 2 mg still needed to be provided by food [53]. Moreover, many foods can increase or decrease inorganic- and heme-iron absorption [54].

Heme-iron rich foods are the best source for human daily iron needs. A rich source of heme-iron is red meat, whose consumption is widespread in Western countries [5]. However, meat contains only 40% of heme-iron, the rest being inorganic iron, absorbed in different ways [55]. Poultry meat and fish are also good sources of heme-iron. Besides, dietary guidelines often suggest fish portions be bigger, therefore providing great amounts of iron as well as omega-3 fatty acids that may stimulate iron metabolism [56]. Moreover, patients with IBD are made aware that red meat may increase the risk of colon cancer, so nutritionists advise those people to shift animal meat consumptions by increasing fish consumption.

Heme-iron is absorbed in the intestine by the enterocyte carrier HCP1 (heme carrier protein 1) [57] and subsequently dissociated from the porphyrinic ring by the HMOX1 (Heme oxygenase 1) protein, then it is stored into ferritin or transported into the bloodstream via the FLVCR1 (Feline leukemia virus subgroup C receptor 1) transporter, where it binds to transferrin (Figure 1B) [58]. Heme-iron, although easier to absorb, is subjected to saturation in a dose-dependent manner. Heme-iron ingested barely under 20 mg can saturate its receptor on the enterocytes and drastically reduce its absorption rate, compared to inorganic iron, which gets absorbed without any issue way over 20 mg of ingested FeSO_4_ (Ferrous sulfate) [59].

Excellent sources of heme-iron are game meat, liver, spleen, and shellfish [60,61]. Cysteine present in meat and fish increases inorganic iron absorption rates from vegetables up to two- to three-fold its baseline [62].

Non-heme iron, by contrast, is found in plant-based foods [63] and its absorption rate is very low, ranging from 2 to 20% of total iron content [64]. The reasons behind the low absorption rate are the short portion of the intestine with an ideal acid environment for iron solubility and consequent absorption [65], as well as iron uptake inhibition mediated by plant dietary compounds, like phytates and tannins [66]. Nevertheless, a vegetarian dietary pattern offers great amounts of inorganic iron [17], commonly present in ferric (Fe^3+^) form that needs to be reduced to ferrous (Fe^2+^) ions, to be transported into the duodenal enterocytes via DMT1 (Divalent Metal Transporter 1) and then stored into ferritin or exported through ferroportin [57]. Vitamin C is crucial for this reduction step as it transfers an electron to the luminal Fe^3+^ via cytochrome b (Dcytb) to obtain ferrous ions (Figure 1B) [67]. Furthermore, vegetables and fruits are rich in iron-chelating molecules that reduce iron bioavailability [68,69] but can also increase iron uptake by mucosal cells [70].

Furthermore, new superfoods are being investigated as sources of highly bioavailable iron. In particular, increased attention has been given to different species of algae which proved to be valid answers to daily iron requirements [71,72].

Bean leaves are rich in iron and can be used as a treatment for iron deficiency. Using a rat model of induced anemia, Martínez-Zavala et al. used a diet enriched in bean leaves to recover the correct blood cell population percentage [73].

## 3. Nutritional Sources that Hinder Iron Absorption

Other minerals may reduce iron absorption as iron ions compete with other divalent ions for the cellular transporter (Ca^2+^ and Zn^2+^) [74]. Zinc showed inhibitor capabilities when administered with iron to healthy volunteers [75]. Thus, it becomes important to account for inorganic ions concentrations when preparing fortified foods. Recently, an Indian study observed that calcium and iron-fortified milk products were not able to provide the correct amount of iron necessary to improve IDA for children unless they were supplemented with ascorbic acid, thus favoring the absorption of iron itself [9,76]. Staple foods like milk, indeed, contain phosphoproteins, mainly alpha-casein (absent in breast milk [77]) and beta-casein that can chelate iron ions. The first proved to be unattackable by intestinal phosphatases, thus inhibiting iron absorption, whereas the latter can be easily digested and liberates iron ions which are quickly absorbed by the mucosal cells [78]. Thus said, excessive cow milk consumption was investigated as a possible cause for child ID, [79,80] raising great concern in the pediatrics community [81,82].

Eggs contain phosphoproteins too [83,84]. Egg yolks are indeed rich in phosvitin and other proteins that chelate iron very similarly to what casein does; in a study on intestinal iron absorption in rats, egg yolks reduced iron and other micronutrient absorption rates because these proteins are resistant to proteolysis [85]. Conversely, ovalbumin contained in egg whites [86] (even though lacking the great amounts of iron present in egg yolks [87]) when added to diets of IDA patients provided high amounts of bioavailable iron that recovered those patients from their disease [88]. On the other hand, many proteins of vegetable origin do not affect iron absorption. Only soybean proteins showed a reduction in heme-iron absorption rates in a study conducted on 15 healthy female subjects [89]. Conversely, inorganic iron absorption is not influenced by soybean and its derivates consumption [90].

Phosphate ions can chelate iron and reduce its absorption rates. Many processed foods have polyphosphates as additives; they serve to adjust pH, change the ionic environment, and function as bacteriostatic [91,92]; plants are rich in phosphate in the form of phytate too. Phosphate ions are absorbed in the small intestine by a Na^+^-dependent cotransporter and by passive diffusion [93,94]. High phosphate intake, derived from the consumption of foods rich in additives [95,96] or from initial chronic kidney disease (CDK) [97,98] is a real threat that often passes undiscovered and results in hyperphosphatemia. Increased phosphate concentrations can affect iron metabolism both before its absorption and in the bloodstream; this anion can efficiently chelate iron ions as proved in a recent experiment where it deprived bacteria of iron ions [99]. Either way, phosphate and polyphosphate complexes can affect the concentration of iron that is needed for correct erythropoiesis, thus leading to anemia. Hyperphosphatemia, CDK, and anemia are often associated and can show, among the many symptoms, systemic inflammation, and increased kidney damage [100,101]. Interestingly, ferric complexes like ferric citrate proved useful in contrasting hyperphosphatemia and provided great amounts of iron to CDK patients [102,103].

Quercetin, a polyphenol present in red onion, apples, honey, raspberries, red grapes and green leafy vegetable, affects cellular iron content bioavailability, by acting as iron-chelator molecule. Quercetin exposure favors extracellular iron export from dendritic cells, upregulating ferroportin, leading to anti-inflammatory effects and tissue repair program [104,105,106,107,108]. Iron administration to the culture medium overturns this effect and sustains the inflammatory response initiated by lipopolysaccharides (LPS) stimulation [69,107]. Furthermore, quercetin aglycone reduces ferric iron and chelates ferrous iron in the intestinal lumen and gets internalized by GLUT (glucose transporter) receptors on enterocytes, thus favoring inorganic iron absorption [109,110]. Quercetin’s role in iron absorption, however, is still debatable as quercetin reduces ferroportin activity and, consequently, iron efflux to the bloodstream. This, in turn, induces an accumulation of quercetin-iron complexes in the mucosa that are lost daily by mucosal exfoliation [111,112]. Many iron chelators show anti-inflammatory activities because they can deprive immune cells of iron as well as block its uptake by invading bacteria. This dual-edge behavior of iron chelators influences current therapies for anemia and chronic inflammatory diseases, implying that a correct balance between iron supplementation and chelation therapy is needed [113,114,115]. Furthermore, iron-chelating polyphenols may act differently in different tracts of the intestine, depending on the pH. Unabsorbed inorganic iron in the acid environment of the duodenum may be efficiently sequestrated in the colon, ideally dampening bacterial growth (Figure 1A) [116,117].

Foods rich in unsaturated fatty acids, like extra virgin olive oil, even if correlated to benefic effects in different pathological conditions [118,119,120], seem to produce negative effects on iron absorption. A study conducted on rats showed that unsaturated and polyunsaturated fatty acids influence negatively iron bioavailability, its absorption, and its utilization by the animals when compared to saturated fatty acids [121].

## 4. Nutritional Resources for Iron Management

As aforementioned, nutrition is the only source of iron intake, but numerous nutritional compounds can favor or inhibit iron intake.

Phytate is the main source of plant phosphate ions and it is found in unprocessed cereals and vegetables. It can chelate cations, thus blocking their absorption in the intestinal tract. Humans, however, lack the enzymes needed to digest phytate that possesses many beneficial effects on the intestinal environment in this form, such as anti-inflammatory and anti-cancer ones. Despite this, it can also chelate iron with detrimental effects on general health. Food processed grains, cooking, and fermentation techniques are some ways that can help reducing phytate concentrations in foods [122].

Milk of animal origin that contains phosphoproteins can reduce inorganic iron absorption. Vegetarians and vegans often consume soymilk or other vegetable-based kinds of milk; in particular, soymilk was compared to iron-fortified cow milk given to infants and a group of breastfed children. This study concluded that soymilk and iron-fortified cow milk were able to prevent anemia in those young, despite the lower amount of inorganic iron contained in soymilk [123]. Similar results were obtained when comparing intestinal iron absorption derived from cow milk or soymilk in weaning rats [124].

Organic acids, too, can increase iron bioavailability. Tartaric, malic, succinic and fumaric acids enhance ferrous and ferric iron uptake. Citric and oxalic acid, on the other hand, decrease ferrous iron uptake but increase ferric iron uptake. Citric acid can increase inorganic iron absorption similarly to ascorbic acid too; for this reason, fortification formulations are employing iron-citrate compounds to provide greater amounts of iron to people in need [13,70]. For example, it has been observed that citric acid added to iron-fortified biscuits effectively increased iron bioavailability [125].

Nowadays, iron-fortified foods include milk, cereals, and beverages [8,9,126,127]. These fortified foods help healthy people meet daily iron requirements.

Moreover, propionic and acetic acid raises ferrous iron uptake only. Salovaara and her team studied the effects of pH, carboxylic, and hydroxyl groups of these organic acids on iron absorption by Caco2 cells. Their results proved that the pH lowered by those acids and, more importantly, ferric iron-organic acid chelates sharply increased inorganic iron bioavailability [128,129]. All these important organic acids are diffuse in many fruits and herbs [129,130,131,132], thereby reinforcing the need for plant-based products in everyday meals [133,134].

Legumes possess high amounts of inorganic iron [135,136] yet difficult to absorb because of their also high content of phytates and tannins [137]. The addition of acid substances, processing, and biofortification of legumes are in the spotlight as potential strategies that could improve iron bioavailability and absorption, to transform these often forgotten and little consumed seeds [138,139]. The addition of lemon juice to hummus has, by far, greatly increased Caco2 iron absorption rates compared to cooked chickpeas alone [140].

Not surprisingly, meat and its heme-iron content can induce a marked increase in inorganic iron bioavailability. A study conducted on weaning children found that adding small quantities of meat to vegetable puree raised the amount of iron in their blood; moreover, meat added to a mixture of legumes and orange juice produced large amounts of absorbable inorganic iron [141,142,143].

Many nutritional resources and food-processing expedients are available to increase inorganic iron availability and they only need to be applied by nutritionists and physicians to overcome ID derived from low consumption of the above-mentioned foods.

Commercially available food additives and fortified foods, however, are not always successful nor accessible to IDA patients or subjects whose dietary intake of iron is far below the recommended levels. Research on new foods that contain easily absorbable iron and that exert a protective role against inflammation is therefore gaining against.

## 5. Superfoods as Nutritional Strategies for Iron Level Replenishment

Newly discovered nutritional strategies employ foods or additives that derive from the so-called “superfoods”, which do not have any scientifically based or regulated definition but generally are considered so when they provide high levels of desirable nutrients, proven to be promising in the prevention of a disease, or believed to offer health benefits [51].

The term appeared in the early part of the 20th Century as a strategy to market bananas. Bananas consumption used to be promoted as a daily source of cheap, easily digestible nutritious food. With the increasing popularity of this fruit, its moniker began to circulate in the public; physicians endorsed bananas to treat lots of ailments, including celiac disease, electrolyte imbalances, etc. [144].

Nowadays, foods that possess high concentrations of nutrients, limited caloric content and show antioxidant and anti-inflammatory properties are often called superfoods by nutritionists and media [145,146]; nevertheless, they are often overlooked on food stores shelves. Some examples include foods like milk rich in calcium and minerals derived from unconventional animals like camel [147] and donkey [148,149], berries [150] and scarcely cultivated vegetables or vegetable parts that are discarded during food processing [73], indigenous vegetables [151] sustainable, and nutritious diets, as well as omega-3 fatty acids from fish or nuts. The potential benefits of superfoods have great margins of employment in the treatment of chronic inflammation [48,152] or as supplements in healthy dietary patterns [153]. Among them, some superfoods contain significant amounts of iron, thus being useful as supplements for people with ID.

Staple foods like meat, legumes, and grain products need iron fortification, correct processing, or the right supplementation to provide the right amounts of iron needed by people. ID patients or people living in underdeveloped countries, despite all these available nutritional strategies, still need way larger amounts of iron to recover from their condition [154].

Thus, research is focused on formulating food supplements that allow a reduction of unabsorbed intestinal iron and its consequent deleterious effects on the mucosa; moreover, recent studies investigated formulations able to enhance non-heme iron absorption.

New approaches to cure ID employ probiotics, polysaccharide-iron complexes, and liposomal iron. Besides, superfoods like algae and iron-enriched grains are vastly studied in low-income countries (Table 1).

Amon the plethora of so-called superfoods only few of them combine high iron content and anti-inflammatory properties. It is important, indeed, to consider the anti-inflammatory potential of those foods to compensate for possible deleterious effects of high amounts of inorganic iron on the intestinal tract [155,156,157]. Thus, the advantage of administrating balanced supplements releasing iron to the proximal part of the small intestine and, at the same time, provide anti-inflammatory and protective compounds. Therefore, we discuss in this section about some of the most interesting superfoods currently available.

In many studies, Lactobacillus plantarum has proved to be a successful probiotic strain in enhancing dietary iron absorption; during iron sulfate therapy its freeze-dried formulation also performed better and had increased stability and vitality over time [158,159,160]. Another mixture of freeze-dried probiotic bacteria (Bifidobacterium bifidum W23, Bifidobacterium lactis W51, Bifidobacterium lactis W52, Lactobacillus acidophilus W37, Lactobacillus brevis W63, Lactobacillus casei W56, Lactobacillus salivarius W24, Lactococcus lactis W19, and Lactococcus lactis W58) has been used to study the absorption rates of iron and other metals from rat standard diet; liver iron accumulation increased significantly as well as hemoglobin parameters, indicating a positive effect on rat iron status [160]. In conclusion, probiotics can increase iron absorption by approximately 50% as seen with a fruit drink already enriched with iron; moreover, they can reduce colonic inflammation in murine models of IL-10 knockout by decreasing mucosal IL-12, IFN-γ and IgG_2a_ levels [190]. Importantly, IL-10 knockout mice can mimic a population of IBD patients that fail to respond to pharmacological therapies, thus considered even more fragile [191].

Several algae have been studied for their potential beneficial effects as iron sources, thus they are considered superfoods. Among them, the Mankai alga (also known as duckweed) was investigated as a potential iron supplement source in a rat model of anemia. After six months of Mankai enriched diet, the physiological levels of hemoglobin and normal blood parameters were restored [162,163].

Of note, iron content in several species of algae is cyclical. Macroalgae belonging to the genera of Ulva, Sargassum, and Porphyria, possess the highest iron contents during spring (even exceeding human daily requirements) and reduced levels of algae are harvested during different seasons. Nevertheless, iron can be better assimilated if compared to other sources of inorganic iron, likely due to high vitamin content [71,72,162].

Ulva polysaccharides conjugated with iron ions can effectively rescue mice from artificially induced anemia [165]. Moreover, Ulva polysaccharides-iron molecules raised B and T cell levels to a number comparable to control animals [166]. Microalgae like Tetraselmis sp. CPT4, Spirulina, and Chlorella were tested for their nutritional components and proved to be rich in iron and antioxidant molecules as well as vitamins and other micronutrients that are essential to humans [167,192]. Tetraselmis sp. CPT4 has recently been produced in large-scale bioreactors and its nutritional profile resulted in biomass richer in iron and many other components (amino acids, vitamins, fibers, and antioxidants) when compared to other microalgae like Arthrospira sp. and Chlorella sp.; its alcoholic extracts showed good ferric reducing and radical scavenging potential. Microbiological and toxicological analyses did not show any potential threat for the employ of this microalga in nutrition [168].

Similarly, algae belonging to the genus Gracilaria were analyzed and proved to contain great amounts of bioactive components and inorganic iron; its extracts were able to reduce inflammatory cytokine production and cancer cell growth in vitro [169].

Moreover, algae can fight inflammation as they are rich in many bioactive compounds that can decrease immune cell activity in vitro and in vivo [170].

Many mushrooms contain polysaccharides that can be easily chelated with iron ions. Naturally, these polysaccharides showed interesting anti-inflammatory and antioxidant properties as well as immune-modulating effects [171,172,193]; in combination with iron particles, they can bypass all the oral iron therapy-related side effects on the gastrointestinal system. A Grifola frondosa iron conjugate showed important immune-modulating activity while increasing lymphocyte proliferation rates; moreover, it could release high amounts of iron when exposed to artificial gastric juices, mimicking the duodenal environment where iron is physiologically absorbed [173]. Similarly, Auricularia auricularia complexes induced anti-inflammatory and antioxidant effects while improving blood parameters in a rat model of anemia [175]. He et al. obtained the same positive results with oligosaccharides derived from agar and chelated with iron ions [194]. Aspergillus oryzae contains huge amounts of iron and was studied in comparison with FeSO_4_. It proved to be available and easy to absorb, while its bioavailability was higher than FeSO_4_, with a long iron release time [176,177]. Despite its lower iron content, Ganoderma lucidum has a long story behind its fame as a health promoter [195]. Its orally given extracts improved hematological parameters in healthy rats; researchers saw a slight but significant increase in hemoglobin levels and a great rise in leukocyte numbers [196]. This could be probably explained by a combination of positive effects of Ganoderma iron content and its antioxidant and anti-inflammatory properties [197,198].

There are other vegetable sources of iron that are currently being investigated, all of them combine their great iron content with good beneficial antioxidant abilities. Amaranth, Colocasia esculenta, and cowpea leaves were recently rediscovered as potential iron-rich and antioxidant foods that can provide great benefits to patients, such as IBD patients, with low iron levels and high inflammatory status in their intestine [179,180,181].

On the same note as soymilk, soybean leaves contain large amounts of iron (both chelated by phytates and phosphates) that resulted in help in increasing red blood cell iron content in borderline ID women when compared to ferrous sulfate. Soybean leaves were prepared as muffins or soups, to reduce phytate content and free the micronutrients bond to it [182,199].

Breed selection and GM-crops are also revolutionizing the concept of superfoods. Cereals are commonly rich of iron poorly absorbable due to their polyphenol content. New cereal varieties, bred into iron-fortified crops, are characterized by increased iron absorption rates [183,185,200].

Wheat and rice are among the most engineered crops, but their phytate content reduces drastically iron bioavailability of the unprocessed grains; thus, the low employ for these GM crops in third world countries [184]. Cowpea leaves have high iron content; however, it has been fortified to increase amounts of iron in the beans too [186]. Tubers like cassava and potatoes have been iron-fortified too [187,188]; sweet potatoes, indeed, showed a 2-fold increase in the level of bioavailable iron [189].

GM iron fortified crops are an emerging frontier in the fight against ID in many parts of the world, there is increasing interest in endemic crops that can be fortified with iron and other micronutrients to help increase products value and people health.

Remarkably, all these aforementioned foods are not yet available in food and general stores all over the world as their studies are still ongoing. Nevertheless, most of them are diffuse in some regions of the world (mainly Asian countries) where they are consumed in traditional dishes [201,202,203]. Our hope is that, in the next years, formulations based on these products will become available all over the world. Superfood-based supplements will possess high concentrations of iron and other beneficial compounds that can thus be easily used to fortify other foods like cereals or beverages. When they become diffuse in stores and pharmacies they could be recommended by nutritionists to people at risk of developing ID or with ongoing ID as therapies to restore the normal values of hemoglobin and serum transferrin saturation.

## 6. Discussion

ID is widespread both in developing and developed countries, particularly affecting women and children and in westernized countries for patients affected by IBD-caused blood loss. Iron storages and red blood cell reserves can sustain most of the daily requirements with only a little amount of metal needed. Iron is taken up from various nutrients by enterocytes located in the first tract of the small intestine, the duodenum, and its absorption is tightly regulated by body storages through hepcidin and ferroportin expressions. Even though the body senses a shortage of iron and its absorptive mechanisms are functional, several food components can interfere with the iron assimilation process, chelating it and preventing its absorption.

Research on ID focused a lot on third world countries, where heme-iron rich food sources are rare, intake is discontinuous, and diets are mainly based on vegetables. Vegetables are, indeed, a great source of iron-chelating molecules that reduce iron absorption. However, iron-chelate complexes derived from algae and plants can provide huge amounts of absorbable iron.

To overcome ID, many different therapies are available, but none is side effects free; therefore, the scientific community is challenged to find more effective strategies that are less likely to cause side effects. To this line, important findings could come from animal models especially in the case of chronic intestinal inflammation and its related complications thanks to their ability to better resemble the human pathology [192,193,194].

Iron is present in many foods but only few of them possess high concentrations of easily absorbable iron, and they are not consumed on regular basis and sufficient quantities. For these reasons iron supplements are becoming extremely diffused. Iron fortification of food is somehow providing interesting results in the fight for ID-derived from malnutrition; however, many studies are based only on infants [204,205]. Adults or patients with other diseases like IBD, need higher amounts of iron and can benefit from other micronutrients that are present in superfoods, particularly phytochemicals with anti-inflammatory activity.

Superfoods are one of the new frontiers explored during recent years to impact at the same time iron homeostasis and immune function. Single bioactive compounds found in superfoods can be thereby studied [206,207] and used as personalized adjuvant medicine, where new targets get discovered day by day. We predict that new combinations of superfoods, with high iron content, antioxidant, and anti-inflammatory effects will be sold in many stores and prescribed as supplements in personalized diets and therapies. Interestingly, these anti-inflammatory properties can be useful in the fight against latent inflammation in NCDs. Recovery from the disease (whether through bariatric surgery [208] or dietary approaches [209]) can quickly restore the correct iron status in these patients. Superfoods can, indeed, be of help in this fight because vitamins alone cannot increase iron absorption in obese women, thus the need for new foods and supplements that can provide iron and protect our body from inflammation [210,211,212].

Superfoods can be efficiently added to many different healthy dietary patterns [213], from vegan to omnivorous, as whole, or as processed supplements to fortify foods items, positively influencing health and promoting the prevention of common chronic degenerative diseases. Indeed, a good cost-effectiveness ratio has been shown providing utility in terms of health to people purchasing these products [214]. Moreover, production and marketing of such products has raised, and consumption is increasingly common [153]. It will not be a surprise to see new iron supplements as superfoods or superfood-derived supplements on market shelves in the years to come.

At the current state of the investigation, these novel nutritional strategies seem to perform better than commonly used therapies, showing no side effects and great bioavailability, but they still need further improvements. Given all these potential benefits, it is auspicial that food formulations based on these newly described plants, algae, and probiotics or diets comprising these foods will be soon tested in vivo and then for the treatment of ID. In the next years, we believe that iron-rich foods and supplements derived from them will become a staple, not only in developing countries but also in food stores as well as being sold in pharmacies.

## Figures and Tables

**Table 1:** Categories of superfoods with high iron content and their effects on health.

Category of Superfood	Beneficial Effects	References
Probiotics	Increase of iron absorption rates	[158,159,160,161]
Algae	Increase of iron absorption rates Increase of hemoglobin levels Antioxidant and anti-inflammatory	[162,163,164,165,166,167,168,169,170]
Mushrooms	Easy-to-absorb iron Anti-inflammatory Immunomodulatory	[171,172,173,174,175,176,177]
Vegetable leaves	High iron content Anti-inflammatory	[178,179,180,181,182]
Fortified crops	Increased iron content Increase of iron absorption rates Anti-inflammatory	[183,184,185,186,187,188,189]
